# Optimal statin use for prevention of sepsis in type 2 diabetes mellitus

**DOI:** 10.1186/s13098-023-01041-w

**Published:** 2023-04-19

**Authors:** Mingyang Sun, Yuan Tao, Wan-Ming Chen, Szu-Yuan Wu, Jiaqiang Zhang

**Affiliations:** 1 Department of Anesthesiology and Perioperative Medicine, Zhengzhou University People’s Hospital, Henan University People’s Hospital, Henan Provincial People’s Hospital, Zhengzhou, China; 2 Graduate Institute of Business Administration, College of Management, Fu Jen Catholic University, Taipei, Taiwan; 3 Artificial Intelligence Development Center, Fu Jen Catholic University, Taipei, Taiwan; 4 Department of Food Nutrition and Health Biotechnology, College of Medical and Health Science, Asia University, Taichung, Taiwan; 5 Division of Radiation Oncology, Lo-Hsu Medical Foundation, Lotung Poh-Ai Hospital, Yilan, Taiwan; 6 Big Data Center, Lo-Hsu Medical Foundation, Lotung Poh-Ai Hospital, No. 83, Nanchang St., Luodong Township, Yilan County, 265 Taiwan; 7 Department of Healthcare Administration, College of Medical and Health Science, Asia University, Taichung, Taiwan; 8 Cancer Center, Lo-Hsu Medical Foundation, Lotung Poh-Ai Hospital, Yilan, Taiwan; 9 Centers for Regional Anesthesia and Pain Medicine, Taipei Municipal Wan Fang Hospital, Taipei Medical University, Taipei, Taiwan; 10 Department of Management, College of Management, Fo Guang University, Yilan, Taiwan

**Keywords:** T2DM, Dose-dependent, Statins, Intensity, Sepsis

## Abstract

**Purpose:**

To investigate the dose-dependent protective effects of statins, specific classes of statins, and different intensities of statin use on sepsis risk in patients with type 2 diabetes mellitus (T2DM).

**Methods:**

We included patients with T2DM aged  ≥ 40 years. Statin use was defined as the use of statin on most days for  > 1 months with a mean statin dose of  ≥ 28 cumulative defined daily doses (cDDDs) per year (cDDD-year). An inverse probability of treatment-weighted Cox hazard model was used to investigate the effects of statin use on sepsis and septic shock while considering statin use status as a time-dependent variable.

**Results:**

From 2008 to 2020, a total of 812 420 patients were diagnosed as having T2DM. Among these patients, 118,765 (27.79%) statin nonusers and 50 804 (12.03%) statin users developed sepsis. Septic shock occurred in 42,755 (10.39%) individuals who did not use statins and 16,765 (4.18%) individuals who used statins. Overall, statin users had a lower prevalence of sepsis than did nonusers. The adjusted hazard ratio (aHR) of statin use was 0.37 (95% CI 0.35, 0.38) for sepsis compared with no statin use. Compared with the patients not using statins, those using different classes of statins exhibited a more significant reduction in sepsis, with aHRs (95% CIs) of sepsis being 0.09 (0.05, 0.14), 0.32 (0.31, 0.34), 0.34 (0.32, 0.36), 0.35 (0.32, 0.37), 0.37 (0.34, 0.39), 0.42 (0.38, 0.44), and 0.54 (0.51, 0.56) for pitavastatin, pravastatin, rosuvastatin, atorvastatin, simvastatin, fluvastatin, and lovastatin use, respectively. In the patients with different cDDD-years of statins, multivariate analysis indicated a significant reduction in sepsis, with aHRs of 0.53 (0.52, 0.57), 0.40 (0.39, 0.43), 0.29 (0.27, 0.30), and 0.17 (0.15, 0.19) for Q1, Q2, Q3, and Q4 cDDD-years (*P* for trend < 0.0001). The optimal daily statin dose of 0.84 DDD was associated with the lowest aHR. Similar trends of higher cDDD-year and specific statin types use were associated with a decrease in septic shock when compared to statin non-users.

**Conclusion:**

Our real-world evidence demonstrated that the persistent use of statins reduced sepsis and septic shock risk in patients with T2DM and a higher cDDD-year of statin use was associated with an increased reduction of sepsis and septic shock risk in these patients.

**Supplementary Information:**

The online version contains supplementary material available at 10.1186/s13098-023-01041-w.

## Keypoints

**
Question**: Is any real-world evidence of the dose-dependent protective effects of the use of specific classes and intensities of statins on sepsis in type 2 diabetes mellitus (T2DM) available?

**Findings**: Our study demonstrated that the persistent use of statins (≥28 cumulative defined daily doses per year [cDDD-year]) reduced sepsis risk in T2DM. A higher cDDD-year of statins was associated with greater reduction of sepsis risk in patients with T2DM. Pitavastatin exerted the strongest protective effect on mortality, followed by pravastatin, rosuvastatin, atorvastatin, simvastatin, fluvastatin, and lovastatin. The optimal daily statin dose of 0.84 DDD was associated with the lowest sepsis risk.

**Meaning**: This is the first study to demonstrate the dose- and intensity-dependent protective effects of different classes of statins on sepsis risk in patients with T2DM.

**Supplementary Information:**

The online version contains supplementary material available at 10.1186/s13098-023-01041-w.

## Introduction

Patients with diabetes are more likely to have wounds and sores that do not heal and may become infected, leading to sepsis [1]. Over 90% of patients with diabetes have type 2 diabetes mellitus (T2DM), which affects hundreds of millions of individuals worldwide [2]. T2DM is characterized by hyperglycemia, insulin resistance, impaired insulin secretion, and dyslipidemia (high triglyceride levels and low high-density lipoprotein cholesterol level) [3–6]. Moreover, diabetes alters the immune system, resulting in an increased risk of sepsis [1]. T2DM is associated with increased risks of recurrent, nosocomial, and secondary infections that lead to sepsis [1, 7]. Patients with T2DM have a higher risk of community-acquired pneumonia, biliary disease, cutaneous infections, and aspiration pneumonia during hospitalization [1, 8]. Patients with T2DM undergoing surgery may have a high risk of infectious complications that lead to sepsis, ventilator-associated pneumonia, and central venous catheter–related infections [1, 8–10].

Many studies evaluating the association between statin use and sepsis in different populations and at various endpoints for statin use have reported controversial findings [11–20]. In terms of sepsis prevention, statin users had superior outcomes than did nonstatin users [19, 20]. However, in severely ill hospitalized patients with diseases such as pneumonia, statin use did not prevent mortality or sepsis-related mortality [11–18]. Statins might prevent diseases through various mechanisms, such as by reducing the cholesterol level and exhibiting anti-inflammatory, immunomodulatory, antioxidant, antithrombotic, and endothelium-stabilizing properties [19–23]. The inconsistency in the aforementioned findings may be attributable to the slow effects of statins. Thus, statin use might prevent the progression of diseases, such as cardiovascular disease [24, 25], stroke [26, 27], and mortality [28], only in relatively healthy individuals instead of severely ill hospitalized patients with several diseases. Moreover, the inconsistent findings may be attributable to the inclusion of different populations and various endpoints for statin use [11–20]. The use of statins as a preventive medication can be beneficial in specific populations, especially patients with T2DM with a high prevalence of inflammatory diseases, immune disorders, oxidative stress conditions, and thrombotic and endothelial diseases, which lead to a high risk of sepsis [29–31]. A protective, safe, and long-term medication for the prevention of sepsis in the susceptible T2DM population is not yet available.

By using a real-world database, in this study, we investigated the dose-dependent protective effects of statins, specific classes of statin, and different intensities of statin use on sepsis risk in T2DM. In addition, we determined the optimal daily statin dose to prevent sepsis in patients with T2DM.

## Patients and methods

### Study population

We conducted a population-based cohort study by using data from Taiwan’s National Health Insurance (NHI) Research Database (NHIRD). The NHIRD contains all medical claims data regarding the disease diagnoses, procedures, drug prescriptions, demographics, and enrollment profiles of all NHI beneficiaries [32]. The NHIRD is linked by encrypted patient identifiers. In addition, the NHIRD data are linked to the Death Registry to ascertain the vital status and cause of death of each patient.

Our cohort included patients who were diagnosed as having T2DM between 2008 and 2020 and were aged  ≥ 40 years. Patients with missing information on age were excluded. To investigate the protective effects of different classes of statins on sepsis, we excluded patients who used different classes of statins during the follow-up period. Statin use was defined as using statin on most days for  > 1 months within 1 year, with a mean statin dose of  ≥ 28 cumulative defined daily doses (cDDDs) per year (cDDD-year). The index date was the date of statin use (≥ 28 cDDD-year). The observation period for each patient began from the index date and continued until death, hospital admission for sepsis, or the end of the study period (December 31, 2021). For patients with more than one episode of sepsis, we analyzed their first episode. Patients who developed sepsis before the index date were excluded. Patients with T2DM who were prescribed  ≥ 28 cDDD-year of statins with a prescription duration of  > 1 months were included in the case group, and those who were prescribed 0 cDDD of statins during the follow-up period were included in the control group.

Sepsis patients were defined as those who were diagnosed with sepsis for the first time and received antibiotic treatment during their hospitalization, based on the ICD-9-CM and ICD-10-CM code. Patients with recurrent sepsis were excluded from the study. It is important to note that all enrolled sepsis patients had no prior history of sepsis and were experiencing the condition for the first time. The ICD-9-CM and ICD-10-CM official guidelines specify the use of specific codes for sepsis, severe sepsis, and septic shock. Specifically, the codes "038.xx" and "995.91" are utilized for sepsis, while "995.92" and "758.52" are designated for septic shock. In the ICD-10-CM coding system, the codes "A40.xx" and "A41.xx" are used to identify sepsis, with the fourth digit specifying the organism causing the infection. The code "R65.20" is used for severe sepsis without septic shock, while the codes "R65.21" and "R65.22" are used for septic shock of different severity levels.

### Study covariates

We included other covariates to adjust for potential confounding effects. Patients were divided into the following age groups: 40 to 50, 51 to 60, 61 to 70, and  ≥ 71 years at the index date. To reduce the effects of potential confounders when comparing sepsis between the statin user and nonuser groups, we used the inverse probability of treatment-weighted (IPTW) [33] Cox regression models with adjustment for age groups, sex, income levels, urbanization, types of antidiabetic drugs used, antidiabetic drugs, diabetic severity (adapted Diabetes Complications Severity Index [aDCSI] score), coexisting comorbidities, and the Charlson comorbidity index (CCI) score (Table 1). We used the date of statin use (≥ 28 cDDD-year) as the index date and matched nonstatin users by using variables collected at this index date. Repeat comorbidities were excluded from CCI scores to prevent repetitive adjustment in multivariate analysis. Comorbidities were determined in accordance with *International Classification of Diseases, Ninth Revision, Clinical Modification* and *International Classification of Diseases, Tenth Revision, Clinical Modification* codes in inpatient records or based on whether the number of outpatient visits was ≥ 2 within 1 year. Onset of comorbidities during 1 year prior to the index date was recorded. Continuous variables are presented as the mean ± standard deviation or median (first and third quartiles) where appropriate.

**Table 1:** Baseline characteristics of the overall T2DM cohort by statin use status

Characteristic	Statin nonusers		Statin users		ASMD
	N = 411,489		N = 400,931		
	N	%	N	%	
Age (mean ± SD), y	56.22 ± 20.53		56.92 ± 29.24		
Age, median (IQR), y	55.00 (46.00,67.00)		55.00 (47.00,66.00)		
Age group, y					0.0010
≤ 50	143 677	34.92%	139 482	34.79	
51–60	110 405	26.83	107 862	26.90	
61–70	82 621	20.08	81 887	20.42	
≥ 71	74 786	18.17	71 700	17.88	
Sex					0.0030
Female	192 076	46.68	188 342	46.98	
Male	219 413	53.32	212 589	53.02	
Income levels (NTD)					0.0030
1. Low income	6149	1.49	5892	1.47	
2. Financial dependent	126 994	30.86	124 418	31.03	
3. ≤ 20 000	195 300	47.46	190 132	47.42	
4. 20 001–30 000	38 667	9.40	37 420	9.33	
5. 30 001–45 000	28 013	6.81	27 225	6.79	
6. > 45 000	16 366	3.98	15 844	3.95	
Urbanization					0.0023
Rural	117 296	28.51	113 367	28.28	
Urban	294 193	71.49	287 564	71.72	
Types of antidiabetic drugs used					0.0119
Zero	147 800	35.92	146 233	36.47	
One type	102 976	25.03	100 092	24.96	
Combined two types	103 361	25.12	99 708	24.87	
Combined three types	41 629	10.12	39 869	9.94	
≥ 4 types	15 723	3.82	15 029	3.75	
Antidiabetic drugs					
Insulin	64 028	15.56	40 124	10.01	0.0555
Metformin	174 582	42.43	174 257	43.46	0.0104
SU	196 379	47.72	196 834	49.09	0.0137
AGI	23 855	5.80	25 500	6.36	0.0056
TZD	15 598	3.79	19 607	4.89	0.0110
DPP4i	426	0.10	211	0.05	0.0005
SGLT2i	2936	0.71	3221	0.80	0.0009
Others	23 473	5.70	23 036	5.75	0.0004
Diabetic severity					
aDCSI score (mean ± SD)	0.96 ± 1.84		0.93 ± 1.84		0.0005
Median (IQR, Q1, Q3)	0.00 (0.00,2.00)		0.00 (0.00,2.00)		
aDCSI score					0.0082
0	215 580	52.39	211 500	52.75	
1	87 406	21.24	84 917	21.18	
2	60 944	14.81	58 986	14.71	
≥ 3	47 559	11.56	45 528	11.36	
aDCSI					
Retinopathy	18 838	4.58	23 234	5.79	0.0122
Nephropathy	48 316	11.74	45 488	11.35	0.0040
Neuropathy	38 843	9.44	42 258	10.54	0.0110
Cerebrovascular	43 085	10.47	38 698	9.65	0.0082
Cardiovascular	105 371	25.61	101 720	25.37	0.0024
Peripheral vascular disease	15 988	3.89	14 841	3.70	0.0018
Metabolic	9617	2.34	6965	1.74	0.0060
Coexisting comorbidities related to the risk of sepsis					
Hypertension	192 670	46.82	185 003	46.14	0.0068
Rheumatoid arthritis	12 485	3.03	11 888	2.97	0.0007
Ankylosing spondylitis	6017	1.46	5804	1.45	0.0002
Psoriasis	3055	0.74	2948	0.74	0.0001
Psoriatic arthritis	248	0.06	305	0.08	0.0002
Crohn’s disease	5860	1.42	5618	1.40	0.0002
Ulcerative colitis	875	0.21	870	0.22	0.0000
COPD	78 625	19.11	75 853	18.92	0.0019
Chronic liver disease	93 080	22.62	90 668	22.61	0.0001
Chronic kidney disease	8217	2.00	7731	1.93	0.0007
Heart failure	22 872	5.56	21 821	5.44	0.0012
Coronary artery disease	85 220	20.71	81 780	20.40	0.0031
Stroke	49 549	12.04	47 196	11.77	0.0027
Coagulopathy	677	0.16	644	0.16	0.0000
Dementia	9045	2.20	7958	1.98	0.0021
Psychosis	828	0.20	828	0.21	0.0001
SLE	7212	1.75	7506	1.87	0.0012
AIDS	132	0.03	137	0.03	0.0000
Cancer	20 318	4.94	13 366	3.33	0.0160
Medication use related to the risk of sepsis					
Immunosuppressant	4 156	1.01	4 129	1.03	0.0001
Systemic corticosteroid	6 008	1.46	5 894	1.47	0.0001
CCI scores					
Mean (SD)	0.93 ± 1.82		0.94 ± 1.79		0.0001
Median (Q1-Q3)	0.00 (0.00,2.00)		0.00 (0.00,2.00)		
CCI scores					0.0017
0	225 531	54.81	220 422	54.98	
≥ 1	185 958	45.19	180 509	45.02	
Different classes of statins					
Lipophilic statins					
Atorvastatin	0	0.00	144 241	35.98	
Lovastatin	0	0.00	27 887	6.96	
Simvastatin	0	0.00	79 435	19.81	
Fluvastatin	0	0.00	37 210	9.28	
Pitavastatin	0	0.00	2950	0.74	
Hydrophilic statins					
Rosuvastatin	0	0.00	78 611	19.61	
Pravastatin	0	0.00	30 597	7.63	
Cumulative dose of statins (cDDD per year)					
Q1	0	0.00	116 915	29.16	
Q2	0	0.00	109 402	27.29	
Q3	0	0.00	95 381	23.79	
Q4	0	0.00	79 233	19.76	
DDD					
≤ 1	0	0.00	354 124	88.33	
> 1	0	0.00	46 807	11.67	
					*P* value
Follow-up time					
Mean (SD) follow-up	8.44 ± 2.65		8.48 ± 1.76		.5930
Median (IQR) follow-up	8.65 (7.55,9.78)		8.65 (7.58,9.76)		
Sepsis					< 0.0001
No	308 643	72.21	371 576	87.97	
Yes	118 765	27.79	50 804	12.03	
Septic Shock					< 0.0001
No	368 734	89.61	384 166	95.82	
Yes	42 755	10.39	16 765	4.18	

*ASMD* absolute standardized mean differences, *SD* standard deviation, *IQR* T2DM, type 2 diabetes mellitus, *Q* quartile, *DDD* defined daily dose, *cDDD-year* cumulative defined daily doses per year, *AIDS* acquired immunodeficiency syndrome, *CCI* Charlson comorbidity index, *COPD* chronic obstructive pulmonary disease, *SLE* systemic lupus erythematosus, *y* years old, *NTD* New Taiwan dollar, *aDCSI* adapted Diabetic Complication Severity Index, *SU* Sulfonylureas, *AGI* Alpha glucosidase inhibitors, *TZD* Thiazolidinedione, *DPP4i* Dipeptidyl peptidase 4 inhibitors, *SGLT2i* Sodium-glucose cotransporter-2 inhibitors

### Outcome variables

Development of sepsis was the primary study outcome. Septic shock was identified as the second outcome.

### Exposure to statins

Prescriptions for statins were coded in accordance with the Anatomical Therapeutic Chemical (ATC) coding system of the NHIRD pharmaceutical subsidies and were used as an interface for retrieving pharmaceutical claims data. In accordance with the ATC classification system, we selected lipophilic (atorvastatin, fluvastatin, lovastatin, simvastatin, and pitavastatin) and hydrophilic (pravastatin and rosuvastatin) statins [34] as the major exposures of interest. In addition, we examined the intensity of statin use by continually estimating the average statin dose as the defined daily dose (DDD) divided by total prescription days. The intensity of statin use was divided into average daily doses below or above 1 DDD. Furthermore, we divided patients into four subgroups that were stratified by the quartiles (Q) of cDDD-year. All analyses were adjusted for age groups, sex, income levels, urbanization, types of antidiabetic drugs used, antidiabetic drugs, diabetic severity (aDCSI score), coexisting comorbidities, current smoking, alcohol liver diseases, and CCI scores.

### Statistical analysis

**Fig. 1:**
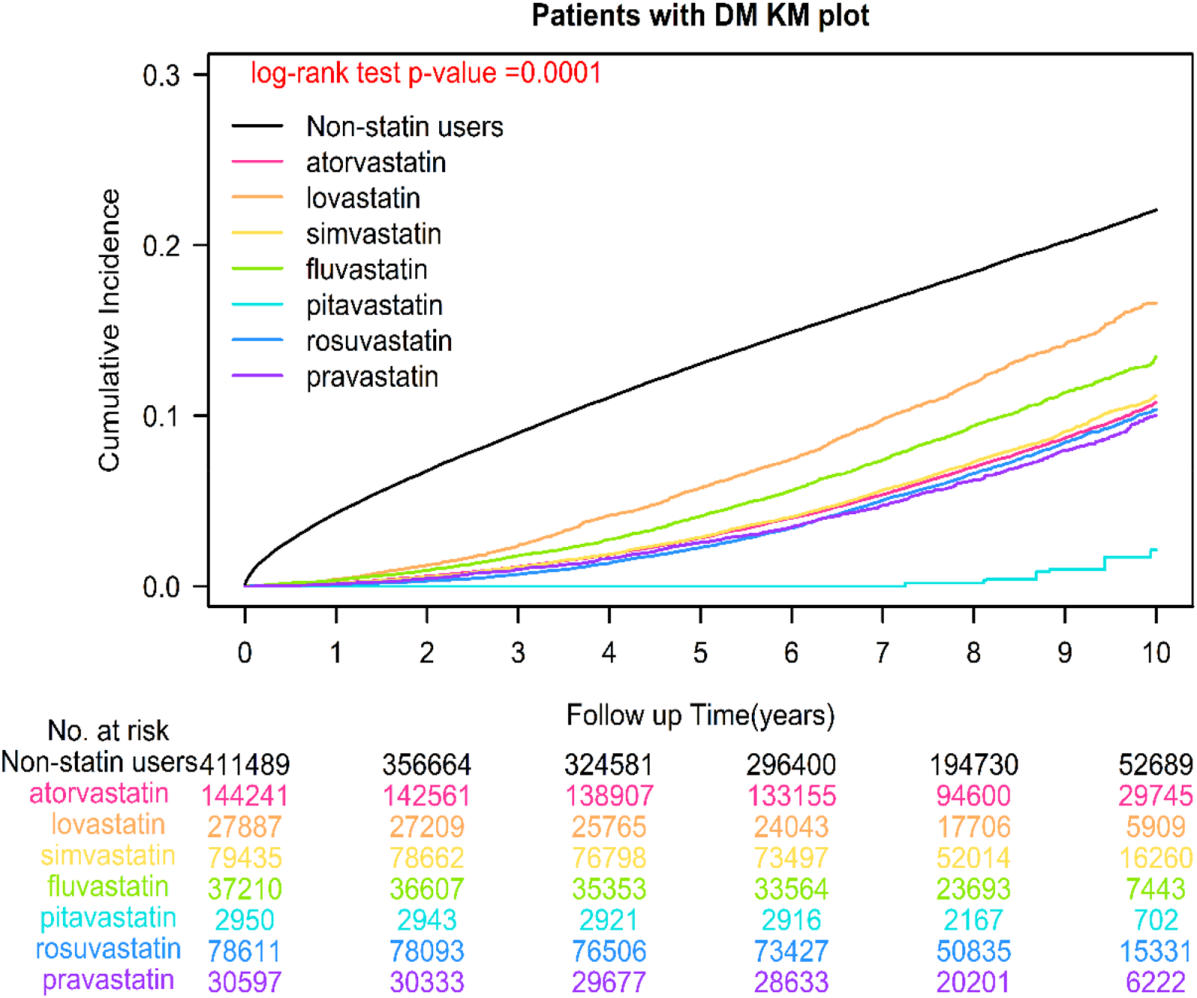
Kaplan–Meier analysis of the cumulative curves of sepsis for different classes of statins in patients with T2DM

**Fig. 2:**
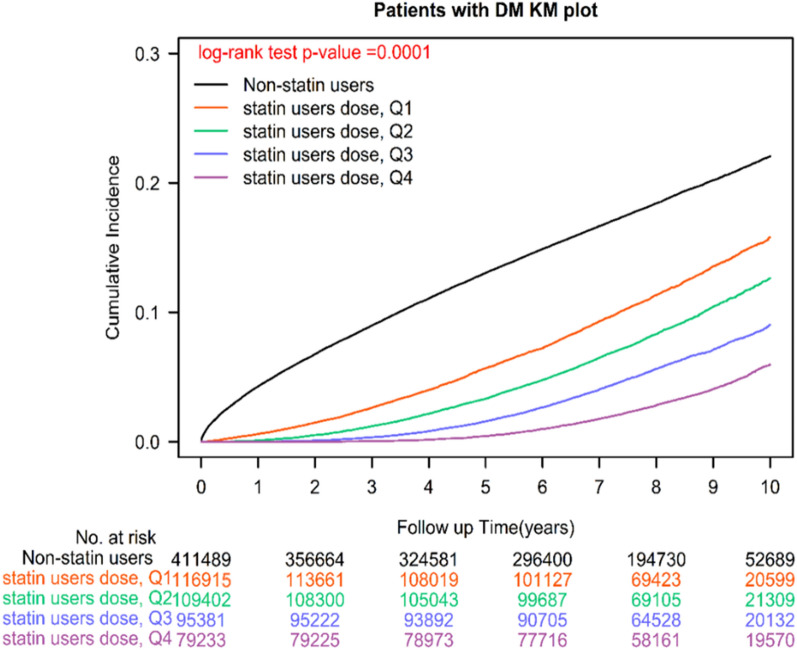
Kaplan**–**Meier analysis of the cumulative curves of sepsis for different cDDD-years of statins in patients with T2DM

The IPTW [33] Cox regression model was used to overcome the imbalance in baseline characteristics between statin and nonstatin users after adjustment for age groups, sex, income levels, urbanization, types of antidiabetic drugs used, antidiabetic drugs, diabetic severity (aDCSI score), coexisting comorbidities, and CCI scores. A time-dependent Cox hazard model was used to compare sepsis between statin and nonstatin users after adjustment for the aforementioned confounding factors. Data on statin prescriptions were collected every 3 months to define a user’s status and were estimated as a time-dependent variable. “Event-free” person-times of users before their first prescription and during the 3-month period without a statin prescription were classified as unexposed follow-up times to prevent bias. In addition, we estimated the effects of individual statins on the risk of sepsis. Analyses were performed in subgroups after adjustment for baseline characteristics by using stratification instead of weighting and postdiagnosis statin use, which yielded similar results. The cumulative incidence of sepsis was estimated using the Kaplan–Meier method, and differences between statin users and nonusers were determined using the stratified log-rank test to compare cumulative incidence curves. Differences between statin users and nonusers at different cDDD-years and for specific statin classes were determined using the stratified log-rank test (Figs. 1 and 2). All statistical analyses were conducted using SAS version. 9.4 (SAS Institute, Cary, NC, USA). The study protocols were reviewed and approved by the Institutional Review Board of Tzu-Chi Medical Foundation (IRB109-015-B).

## Results

From 2008 to 2020, a total of 812 420 patients were diagnosed as having T2DM. The mean age at T2DM diagnosis were 56.22 and 56.92 years for the nonstatin users and statin users, respectively. Furthermore, 35.98% of the statin users received atorvastatin, which was the most prescribed statin, followed by simvastatin (19.81%) and rosuvastatin (19.61%). To ensure postmatch balance, we used the absolute standardized mean difference (ASMD) of < 0.1 after IPTW for all baseline covariates [35]. The ASMDs for all covariates were < 0.1, indicating that the covariates after IPTW were balanced between the statin users and nonusers (Table 1) [35].

### Sepsis, comparison of different classes of statin use, and dose-dependent protective effects

Among the patients with T2DM, 118 765 (27.79%) nonstatin users and 50 804 (12.03%) statin users developed sepsis. Overall, statin users had a lower incidence of sepsis than did the nonusers. The adjusted hazard ratio (aHR) of statin use was 0.37 (95% CI: 0.35, 0.38) for sepsis compared with nonstatin use (Table 2), and the log-rank test yielded a *P* < 0.0001 (Additional file 1: Figure S1). The findings of the Cox regression model revealed that compared with the nonstatin users, those using different classes of statins exhibited a significant reduction in sepsis, with the aHRs (95% CI) of sepsis being 0.09 (0.05, 0.14), 0.32 (0.31, 0.34), 0.34 (0.32, 0.36), 0.35 (0.32, 0.37), 0.37 (0.34, 0.39), 0.42 (0.38, 0.44), and 0.54 (0.51, 0.56), respectively, for pitavastatin, pravastatin, rosuvastatin, atorvastatin, simvastatin, fluvastatin, and lovastatin use, respectively (Table 2). The results of the log-rank test indicated that the cumulative incidence of sepsis significantly differed among the patients using different classes of statins (*P* < 0.0001; Fig. 1). In the patients with different cDDD-years of statins, the findings of multivariate analysis revealed a significant reduction in sepsis, with aHRs of 0.53 (0.52, 0.57), 0.40 (0.39, 0.43), 0.29 (0.27, 0.30), and 0.17 (0.15, 0.19) for Q1, Q2, Q3, and Q4 cDDD-year, respectively (*P* for trend < 0.0001), and the log-rank test yielded a *P* < 0.0001 (Fig. 2).

**Table 2:** Sepsis risk and adjusted hazard ratios (aHRs) associated with statin use among patients with T2DM

	Crude HR (95%CI)		*P* value	Adjusted HR (95%CI)^*^		*P* value
Stain users or nonusers						
Nonusers	Reference					
Statin users	0.35	(0.31, 0.36)	< 0.0001	0.37	(0.35, 0.38)	< 0.0001
Different classes of statins						
Nonusers	Reference					
* Hydrophilic statins*						
Pravastatin	0.30	(0.28, 0.32)	< 0.0001	0.32	(0.31, 0.34)	< 0.0001
Rosuvastatin	0.32	(0.29, 0.33)	< 0.0001	0.34	(0.32, 0.36)	< 0.0001
*Lipophilic statins*						
Pitavastatin	0.07	(0.03, 0.09)	< 0.0001	0.09	(0.05, 0.14)	< 0.0001
Fluvastatin	0.44	(0.41, 0.45)	< 0.0001	0.42	(0.38, 0.44)	< 0.0001
Simvastatin	0.33	(0.31, 0.34)	< 0.0001	0.37	(0.34, 0.39)	< 0.0001
Lovastatin	0.57	(0.52, 0.59)	< 0.0001	0.54	(0.51, 0.56)	< 0.0001
Atorvastatin	0.33	(0.32, 0.34)	< 0.0001	0.35	(0.32, 0.37)	< 0.0001
Cumulative dose of statins DDD per year						
Nonusers	Reference					
Q1	0.55	(0.52, 0.56)	< 0.0001	0.53	(0.52, 0.57)	< 0.0001
Q2	0.37	(0.35, 0.39)	< 0.0001	0.40	(0.39, 0.43)	< 0.0001
Q3	0.24	(0.22, 0.25)	< 0.0001	0.29	(0.27, 0.30)	< 0.0001
Q4	0.15	(0.13, 0.16)	< 0.0001	0.17	(0.15, 0.19)	< 0.0001
*P* for trend			< 0.0001			< 0.0001

*aHR* adjusted hazard ration, *HR* hazard ratio, *CI* confidence interval, *DDD* defined daily dose, *T2DM* type 2 diabetes mellitus, *Q* Quartile

^*^The aHR was derived from the inverse probability-weighted Cox model considering statin use as a time-dependent covariate, and the model was adjusted for age groups, sex, income levels, urbanization, types of antidiabetic drugs used, antidiabetic drugs, diabetic severity (aDCSI score), coexisting comorbidities, medication use, and CCI scores

### Intensity of statin use

The optimal intensity of statin use was 0.84 DDD, which has a lower aHR of sepsis (Additional file 1: Figure S2) than the other DDDs. The protective effects of statins on sepsis exhibited a U-shaped dose–response relationship [36]. The optimal milligram recommendations for different statins use were shown in Additional file 1: Table S1.

### Sensitivity analysis

We examined the intensity of statin use and determined that the patients who received on average both ≤ 1 and > 1 DDD had a decreased risk of sepsis. In addition, we investigated the effect of statins on patients with different comorbidities (CCI ≤ 1), age groups, sex, income levels, urbanization, types of antidiabetic drugs used, antidiabetic drugs, diabetic severity (aDCSI Score), and coexisting comorbidities. Reduction in sepsis risk observed in sensitivity analysis was comparable to that noted in the main analysis (Table 3).

**Table 3:** Sensitivity analyses of the association between statin use and sepsis among patients with T2DM

Subpopulation or exposure	No. of patients	Sepsis			
		No. of Sepsis	aHR*	95% CI	*P* value
Age group, y					
≤ 50	283,159	19,762	0.34	(0.31, 0.35)	< 0.0001
51–60	218,267	21,908	0.35	(0.32, 0.36)	< 0.0001
61–70	164,508	26,945	0.38	(0.36, 0.40)	< 0.0001
≥ 71	146,486	40,870	0.39	(0.35, 0.42)	< 0.0001
Sex					
Female	380,418	51,342	0.37	(0.35, 0.38)	< 0.0001
Male	432,002	58,142	0.36	(0.34, 0.37)	< 0.0001
Income levels (NTD)					
1. Low income	12,041	3265	0.36	(0.31, 0.40)	< 0.0001
2. Financial dependent	251,412	55,673	0.37	(0.35, 0.44)	< 0.0001
3. ≤ 20 000	385,432	5389	0.36	(0.34, 0.40)	< 0.0001
4. 20 001–30 000	76,087	2998	0.38	(0.34, 0.41)	< 0.0001
5. 30 001–45 000	55,238	1357	0.35	(0.30, 0.40)	< 0.0001
6. > 45 000	32,210	40,803	0.41	(0.34, 0.49)	< 0.0001
Urbanization					
Rural	230,663	38,083	0.36	(0.34, 0.37)	< 0.0001
Urban	581,757	71,402	0.37	(0.35, 0.38)	< 0.0001
Types of antidiabetic drugs used					
Zero	294,033	30,139	0.40	(0.36, 0.45)	< 0.0001
One type	203,068	26,415	0.39	(0.36, 0.42)	< 0.0001
Combined two types	203,069	28,121	0.36	(0.35, 0.40)	< 0.0001
Combined three types	81,498	16,909	0.32	(0.29, 0.36)	< 0.0001
≥ 4 types	30,752	7902	0.29	(0.27, 0.34)	< 0.0001
Antidiabetic drugs					
Insulin	104,152	21,872	0.44	(0.40, 0.48)	< 0.0001
Metformin	378,934	68,208	0.28	(0.26, 0.35)	< 0.0001
SU	393,213	82 574	0.37	(0.35, 0.40)	< 0.0001
AGI	49,355	9,874	0.36	(0.33, 0.38)	< 0.0001
TZD	35,201	7,043	0.37	(0.33, 0.39)	< 0.0001
DPP4i	637	144	0.41	(0.31, 0.51)	< 0.0001
SGLT2i	6,157	1,354	0.42	(0.30, 0.49)	< 0.0001
Others	46,509	21,500	0.39	(0.34, 0.41)	< 0.0001
aDCSI score					
0	427,080	38 752	0.40	(0.33, 0.43)	< 0.0001
1	172,323	20,273	0.38	(0.35, 0.40)	< 0.0001
2	119,930	23,183	0.34	(0.32, 0.38)	< 0.0001
≥ 3	93,087	27,276	0.32	(0.30, 0.34)	< 0.0001
CCI score					
0	427,080	38,752	0.38	(0.36, 0.40)	< 0.0001
≥ 1	366,467	61,426	0.34	(0.33, 0.41)	< 0.0001
Coexisting comorbidities					
Hypertension	377,673	65,991	0.37	(0.35, 0.38)	< 0.0001
Rheumatoid arthritis	24,373	4497	0.35	(0.30, 0.39)	< 0.0001
Ankylosing spondylitis	11,821	1905	0.36	(0.30, 0.41)	< 0.0001
Psoriasis	6003	1037	0.32	(0.25, 0.39)	< 0.0001
Psoriatic arthritis	553	134	0.14	(0.06, 0.31)	< 0.0001
Crohn’s disease	11,478	1863	0.35	(0.29, 0.40)	< 0.0001
Ulcerative colitis	1745	287	0.36	(0.23, 0.53)	< 0.0001
COPD	154,478	30,107	0.36	(0.33, 0.38)	< 0.0001
Chronic liver disease	183,748	25,026	0.32	(0.30, 0.36)	< 0.0001
Chronic kidney disease	15,948	5053	0.33	(0.31, 0.36)	< 0.0001
Heart failure	44,693	12,089	0.36	(0.33, 0.38)	< 0.0001
Coronary artery disease	167,000	32,251	0.38	(0.35, 0.40)	< 0.0001
Stroke	96,745	24,094	0.33	(0.31, 0.35)	< 0.0001
Coagulopathy	1321	330	0.17	(0.12, 0.24)	< 0.0001
Dementia	17,003	5743	0.31	(0.27, 0.34)	< 0.0001
Psychosis	1656	372	0.48	(0.34, 0.68)	< 0.0001
Ankylosing spondylitis	11,821	1905	0.34	(0.30, 0.41)	< 0.0001
SLE	14,718	3224	0.32	(0.28, 0.36)	< 0.0001
Cancer	33,684	6,733	0.30	(0.25, 0.39)	< 0.0001
DDD					
≤ 1	764,110	104,395	0.33	(0.32, 0.37)	< 0.0001
> 1	48,309	5089	0.57	(0.45, 0.69)	< 0.0001

*DDD* defined daily dose, *AIDS* acquired immunodeficiency syndrome, *CCI* Charlson comorbidity index, *COPD* chronic obstructive pulmonary disease, *SLE* systemic lupus erythematosus, *NTD* New Taiwan Dollar, *aDCSI* adapted Diabetic Complication Severity Index, *aHR* adjusted hazard ration, *CI* confidence interval, *SU* Sulfonylureas, *AGI* Alpha glucosidase inhibitors, *TZD* Thiazolidinedione, *DPP4i* Dipeptidyl peptidase 4 inhibitors, *SGLT2i* Sodium-glucose cotransporter-2 inhibitors

^*^The aHR was derived from the inverse probability-weighted Cox model considering statin use as a time-dependent covariate, and the model was adjusted for age groups, sex, income levels, urbanization, types of antidiabetic drugs used, antidiabetic drugs, diabetic severity (aDCSI score), coexisting comorbidities, medication use, and CCI scores

### IRs and IRRs for sepsis

Overall, significant IRRs of sepsis risk were obtained for the statin users and nonusers (Table 4). The IRR (95% CI) of sepsis risk in the statin users compared with the nonusers was 0.41 (0.40, 0.41), and the IRs of sepsis risk in the statin users and nonusers were 106.03 and 259.09 per 10,000 person-years, respectively. The IRs of sepsis risk for pitavastatin, pravastatin, rosuvastatin, atorvastatin, simvastatin, fluvastatin, and lovastatin users were 16.80, 90.97, 94.76, 100.67, 104.42, 130.19, and 166.85 per 10,000 person-years, respectively. The IRRs (95% CI) of sepsis risk in the statin users compared with the nonusers were 0.61 (0.60, 0.62), 0.46 (0.45, 0.47), 0.32 (0.31, 0.32), and 0.19 (0.18, 0.20), respectively, for Q1, Q2, Q3, and Q4 cDDD-years.

**Table 4:** IR and IRRs for sepsis

	Events	Person-years	IR (10 000 person-year)	IRR	95%CI for IRR	*P* value
Statin users						
Nonusers	73,618	2,841,369.0	259.09	Ref		
Users	35,866	3,382,581.0	106.03	0.41	(0.40, 0.41)	< 0.0001
Classes of statins						
Nonusers	73,618	2,841,369.0	259.09	Ref		
Atorvastatin	12,279	1,219,721.0	100.67	0.39	(0.38, 0.40)	< 0.0001
Lovastatin	3815	228,654.8	166.85	0.64	(0.62, 0.67)	< 0.0001
Simvastatin	7019	672,179.1	104.42	0.40	(0.39, 0.41)	< 0.0001
Fluvastatin	4032	309,680.1	130.19	0.50	(0.49, 0.52)	< 0.0001
Pitavastatin	44	26,126.8	16.80	0.06	(0.05, 0.09)	< 0.0001
Rosuvastatin	6311	665,990.1	94.76	0.37	(0.36, 0.38)	< 0.0001
Pravastatin	2367	260,228.8	90.97	0.35	(0.34, 0.37)	< 0.0001
Cumulative dose of statins (cDDD-year)						
Nonuser	73,618	2,841,369.0	259.09	Ref		
Statin user dose, Q1	14,781	940,970.8	157.08	0.61	(0.6, 0.62)	< 0.0001
Statin user dose, Q2	10,869	915,054.6	118.78	0.46	(0.045, 0.47)	< 0.0001
Statin user dose, Q3	6724	822,236.7	81.78	0.32	(0.31, 0.32)	< 0.0001
Statin user dose, Q4	3492	704,318.9	49.58	0.19	(0.18, 0.20)	< 0.0001

*DDD* defined daily dose, *IR* incidence rate, *IRR* incidence rate ratio, *Ref.* reference, *CI* confidence interval

### Septic shock, comparison of different classes of statin use, and dose-dependent protective effects

To investigate the protective effects of statins against septic shock among patients with T2DM. Of the total T2DM patients, 42,755 (10.39%) did not use statins, while 16,765 (4.18%) did. Statin users exhibited a significantly lower incidence of septic shock than nonusers. After adjustment for confounding factors, the adjusted hazard ratio (aHR) for septic shock was 0.34 (95% CI 0.33, 0.35) in statin users compared to nonusers (Additional file 1: Table S2). Further analysis revealed that the use of different classes of statins was associated with a significant reduction in sepsis, as indicated by the aHRs (95% CI) of sepsis for pitavastatin, pravastatin, rosuvastatin, atorvastatin, simvastatin, fluvastatin, and lovastatin use of 0.06 (0.04, 0.10), 0.29 (0.28, 0.31), 0.31 (0.30, 0.33), 0.32 (0.31, 0.33), 0.34 (0.33, 0.36), 0.38 (0.36, 0.40), and 0.50 (0.48, 0.53), respectively (Additional file 1: Table S2). Moreover, the protective effect of statins against sepsis was dose-dependent. Patients who used statins with higher cumulative defined daily doses (cDDD-years) had a lower incidence of sepsis, with aHRs of 0.53 (0.52, 0.55), 0.39 (0.38, 0.41), 0.26 (0.24, 0.28), and 0.14 (0.13, 0.15) for the lowest to highest quartile of cDDD-years, respectively (P for trend < 0.0001), according to the findings of the multivariate analysis.

## Discussion

Statins appear to possess beneficial anti-inflammatory properties; for instance, statins can suppress the endotoxin-induced upregulation of Toll-like receptor (TLR)-4 and TLR-2 [7, 37]. Some studies have indicated the preventive effect of statins on sepsis in patients with cardiovascular diseases [19, 20]. By contrast, some studies, including the meta-analyses of randomized trials, have reported no beneficial effects of statin use on mortality or sepsis-related mortality in the hospitalized population with pneumonia or active infection [11–18]. However, these studies have included heterogeneous populations, various endpoints, and different statin classes [7, 11–20]. Moreover, these studies did not indicate a clear DDD, the dose-dependent protective effects of statins on sepsis or mortality, the intensity of statin use, and cDDD-year as well as examine the effects of the continued use or discontinuation of statins. Previous studies have reported vague findings regarding the protective effects of different classes, doses, and intensity of statin use on sepsis in the susceptible population with T2DM with a high risk of sepsis [10, 29]. No study has evaluated whether statin use can prevent sepsis in the susceptible population with T2DM. Because T2DM is an independent risk factor for sepsis and patients with T2DM have a high prevalence of sepsis [9, 10, 29], a safe, effective, and long-term protective medication for sepsis is required. We investigated the dose-dependent protective effects of statins, specific classes of statin, and different intensities of statin use on sepsis risk in patients with T2DM. In addition, we determined the optimal daily statin dose to prevent sepsis in patients with T2DM. Our results demonstrated that the aHR of statin use was 0.37 (95% CI: 0.35, 0.38) for sepsis compared with no statin use. Compared with the patients not using statins, those using different classes of statins exhibited a more significant reduction in sepsis, with aHRs (95% CIs) of sepsis being 0.09 (0.05, 0.14), 0.32 (0.31, 0.34), 0.34 (0.32, 0.36), 0.35 (0.32, 0.37), 0.37 (0.34, 0.39), 0.42 (0.38, 0.44), and 0.54 (0.51, 0.56) for pitavastatin, pravastatin, rosuvastatin, atorvastatin, simvastatin, fluvastatin, and lovastatin use, respectively. In the patients with different cDDD-years of statins, multivariate analysis indicated a significant reduction in sepsis, with aHRs of 0.53 (0.52, 0.57), 0.40 (0.39, 0.43), 0.29 (0.27, 0.30), and 0.17 (0.15, 0.19) for Q1, Q2, Q3, and Q4 cDDD-years (*P* for trend < 0.0001). The optimal daily statin dose of 0.84 DDD was associated with the lowest aHR.The optimal intensity of statin use was 0.84 DDD, which resulted in a lower aHR than did other DDDs. Sensitivity analysis indicated that sepsis risk was significantly decreased in the statin users, regardless of age, sex, income levels, urbanization, types of antidiabetic drugs use, antidiabetic drugs, aDCSI score, coexisting comorbidities, medication use, and CCI scores.

To the best of our knowledge, no study has evaluated the protective effects of different classes of statins on sepsis in patients with T2DM. This is the first study to demonstrate the protective effect of specific statins on sepsis in patients with T2DM. Pitavastatin exerted the strongest protective effect on sepsis, followed by pravastatin, rosuvastatin, atorvastatin, simvastatin, fluvastatin, and lovastatin. Statins exert protective effects possibly by reducing low-density lipoprotein (LDL) and triglyceride levels and increasing the high-density lipoprotein (HDL) level [38–40]. For example, rosuvastatin is more potent than atorvastatin [38, 39], and rosuvastatin is significantly more potent than simvastatin, atorvastatin, fluvastatin, and lovastatin [39, 40]. At the maximal prescribed doses, LDL cholesterol reduction is greater with rosuvastatin than with the aforementioned three statins [39, 40]. The efficacy of the aforementioned four statins in reducing the LDL level is similar to their protective effects on sepsis in patients with T2DM (Table 2 and Fig. 1). Statins alter the HDL cholesterol level (also known as the good cholesterol), typically increasing them, but these effects vary by the class and dose of statins [41]. For example, an increase in the HDL cholesterol level is noted with the increasing doses of simvastatin and rosuvastatin, whereas the increase in the HDL cholesterol level caused by atorvastatin is attenuated at its higher doses [41]. Moreover, rosuvastatin was more effective in reducing the triglyceride level than other statins in patients with hypercholesterolemia [39]. However, the association of the effects of specific statins on LDL, HDL, and triglycerides with sepsis remains unclear. In our current study, the effects of specific statins on LDL, HDL, and triglycerides appeared to be proportional to the protective effect of statins on sepsis in the patients with T2DM (Table 2 and Fig. 1). In addition, pitavastatin, pravastatin, and fluvastatin are less likely to have drug interactions or cause muscle toxicity than some other statins [42, 43]. Fewer pharmacokinetic drug interactions are likely to occur with pravastatin, rosuvastatin, pitavastatin, and fluvastatin because they are not metabolized through CYP3A4 [42, 43]. Patients with T2DM receive many types of medication (Table 1); thus, statins with fewer drug–drug interactions, including pitavastatin and pravastatin, might lead to a balance between effects and toxicities [42, 43]. Although the detailed mechanisms of specific classes of statins and their preventive effects on sepsis remain unclear, statins that result in fewer pharmacokinetic drug interactions, including pitavastatin and pravastatin [42, 43], and exert stronger effects on lowering LDL and triglycerides and increasing HDL, including rosuvastatin [38–40], might be better choices. However, because the sample size of pitavastatin users in our study was small, our findings might be biased. Therefore, future studies should investigate the detailed effects of specific statins on sepsis and their underlying mechanisms.

The effects of statins on LDL, HDL, and triglycerides might differ on the basis of their intensity and daily dose because we observed a U-shaped dose–response relationship for the effect of the daily dose of statins on LDL, HDL, and triglycerides [41, 44]. Thus, the U-shaped dose–response relationship was observed for not only the pharmacological but also toxicological effects of statins (Additional file 1: Figure S2) [36]. Thus, in our study, we observed that an increased daily dose of statins did not result in a better protective effect [45]. This might be the reason for the inconsistency in the findings of previous studies on the association of statin use with sepsis risk [7, 11–20]. This is the first study to demonstrate that the optimal intensity of DDD for statin users was 0.84 DDD, which was associated with a lower risk of sepsis in the T2DM population. The U-shaped dose–response relationship observed for the protective effects of statins on sepsis is in agreement with the findings of previous biological, toxicological, and pharmacological studies [36]. Part of the variability in the response to and side effects of statins may be related to genetic differences in the rate of drug metabolism [46–48]. CYP2D6 is a member of the cytochrome P450 superfamily of drug-oxidizing enzymes. CYP2D6 is functionally absent in 7% of White and African American individuals, and its deficiency is rare among Asian individuals. Asian individuals (mostly those from China, Japan, and Korea) may exhibit greater responses to low doses of statins than do European American individuals [47]. Thus, statin therapy should be started with a lower initial daily dose in Asian individuals than in other groups considering the observed differences in pharmacokinetics [47, 49]. Therefore, our study demonstrated that the optimal intensity of statin daily dose was 0.84 DDD, and this value would be valuable for Asian patients and explain the previous inconsistent findings [7, 11–20]. The optimal milligram recommendations for different statins use were shown in Additional file 1: Table S1.

Different cDDD-years for statins might exert different effects on LDL, HDL, and triglycerides and thus different effects on sepsis risk in patients with T2DM. Therefore, we determined the effects of the cumulative doses of Q1, Q2, Q3, and Q4 cDDD-years on sepsis risk in the patients with T2DM. Our results revealed that the aHRs (95% CIs) of the cDDD-year of Q1, Q2, Q3, and Q4 were 0.53 (0.52, 0.57), 0.40 (0.39, 0.43), 0.29 (0.27, 0.30), and 0.17 (0.15, 0.19; *P* for trend < 0.0001). A higher cDDD-year of statins was associated with an increased reduction of sepsis risk in the patients with T2DM. Our results demonstrated the dose-dependent protective effect of statin use on sepsis in the patients with T2DM.

The strengths of our study is that it included the largest sample size of statin users and examined the effects of the intensity and dose-dependent protective effects of statins on sepsis in the patients with T2DM (Figs. 1 and 2 and Additional file 1: Figures S1 and S2). Compared with the findings of previous studies examining the association of statin use with sepsis in different populations, our study provided more reliable and long-term follow-up real-world evidence to indicate that the persistent use of statins can reduce sepsis risk in patients with T2DM (Tables 2–4). In addition, in terms of the intensity of statin use, the optimal daily statin dose of 0.84 DDD was associated with the lowest sepsis risk (Additional file 1: Figure S2). Moreover, pitavastatin exerted the strongest protective effect on sepsis, followed by pravastatin, rosuvastatin, atorvastatin, simvastatin, fluvastatin, and lovastatin (Table 2 and Fig. 1). This is the first study to investigate the dose-dependent protective effects of statins, specific classes of statins, and different intensities of statin use on sepsis risk in T2DM.

This study has some limitations. First, this study was conducted using a claims database. Laboratory values or lipid profiles were not available. Therefore, we could not evaluate whether changes in lipid profiles following the initiation of statin use were associated with sepsis. Second, we could not completely avoid the possibility that statin users might be a different population compared with nonusers, which might have been an unmeasured confounding factor in our study. We used IPTW to balance the difference in covariates. Several subgroup analyses were conducted to examine potential bias resulting from unmeasured confounders. We examined the effects of statins for different age groups, sex, income levels, urbanization, types of antidiabetic drugs use, antidiabetic drugs, aDCSI Score, coexisting comorbidities, medication use, and CCI scores. The reduction in sepsis with statin use was similar in patients with T2DM in sensitivity analysis. Third, we did not have information on the body mass index and other lifestyle factors at the time of T2DM diagnosis. Therefore, we were unable to evaluate the impact of those factors on sepsis. Fourth, event numbers were small in some of the subgroups of specific statin classes, which limited our statistical power. Finally, our study population was 95% Han Chinese [50], which limits the generalizability of our results to other ethnic groups. The prevalence of statin use was approximately 76.5% in North Americans, 69.9% in Western Europeans, and 60.5% in Asians [51]. Therefore, other ethnicities with higher rates of statin use might have slightly different results. However, some previous studies conducted in different ethnic populations also demonstrated a reduction in sepsis risk associated with statin use.

## Conclusion

Our real-world evidence demonstrated that the persistent use of statins reduced sepsis risk in the patients with T2DM and a higher cDDD-year of statins was associated with more reduction in sepsis risk in these patients. The optimal daily statin dose of 0.84 DDD was associated with the lowest mortality. Moreover, pitavastatin exerted the strongest protective effect on sepsis, followed by pravastatin, rosuvastatin, atorvastatin, simvastatin, fluvastatin, and lovastatin.

## Supplementary Information

**Additional file 1: Table S1**. Transformation of the optimal DDD (lowest hazard ratio of sepsis) to daily milligram recommendations among different statins therapy. **Table S2.** Septic shock risk and adjusted hazard ratios (aHRs) associated with statin use among patients with T2DM. **Figure S1.** Kaplan**–**Meier analysis of the cumulative curves of sepsis for statin users and nonusers among patients with T2DM. **Figure S2.** Intensity of statin use (DDD) and the hazard ratio of sepsis. **Figure S3.** Study flow-chart.

## Data Availability

Data analyzed during the study were provided by a third party. Requests for data should be directed to the provider indicated in the Acknowledgments.

## Abbreviations

aHR: Adjusted hazard ratio
CI: Confidence interval
aDCSI: Adapted diabetes complications severity index
cDDD: Cumulative defined daily dose
DDD: Defined daily dose
IQR: Interquartile range
SD: Standard deviation
N: Number
ASMD: Absolute standardized mean difference
HR: Hazard ratio
aH: Adjusted hazard ratio
CI: Confidence interval
T2DM: Type 2 diabetes mellitus
NHI: National Health Insurance
NHIRD: National Health Insurance Research Database
RCT: Randomized controlled trial
cDDD-year: Cumulative defined daily doses per year
IPTW: Inverse probability of treatment-weighted
ATC: Anatomical Therapeutic Chemical
Q: Quartile
CCI: Charlson Comorbidity Index
LDL: Low-density lipoprotein
HDL: High-density lipoprotein
IR: Incidence rate
IRR: Incidence rate ratio
