# Supplementary material for: Optimal statin use for prevention of sepsis in type 2 diabetes mellitus
Source: Diabetol Metab Syndr. 2023 Apr 19;15:75. doi: 10.1186/s13098-023-01041-w (PMC10114454; doi:10.1186/s13098-023-01041-w)
Supplement: Supplementary file 1 — Additional file 1: Table S1. Transformation of the optimal DDD (lowest hazard ratio of sepsis) to daily milligram recommendations among different statins therapy. Table S2. Septic shock risk and adjusted hazard ratios (aHRs) associated with statin use among patients with T2DM. Figure S1. Kaplan–Meier analysis of the cumulative curves of sepsis for statin users and nonusers among patients with T2DM. Figure S2. Intensity of statin use (DDD) and the hazard ratio of sepsis. Figure S3. Study flow-chart. [file 13098_2023_1041_MOESM1_ESM.docx]

**Supplemental Table 1**. Transformation of the optimal DDD (lowest hazard ratio of sepsis) to daily milligram recommendations among different statins therapy

|  | **ATC CODE** | **Sepsis** |
| --- | --- | --- |
|  |  | Optimal DDD for primary prevention of sepsis : 0.84 DDD |
| **Lipophilic statins** | |  |
| Atorvastatin | C10AA05 | 16.80 milligram |
| Lovastatin | C10AA02 | 37.80 milligram |
| Simvastatin | C10BA02 | 25.20 milligram |
| Fluvastatin | C10AA04 | 50.40 milligram |
| Pitavastatin | C10AA08 | 1.68 milligram |
| **Hydrophilic statins** | |  |
| Rosuvastatin | C10AA07 | 8.40 milligram |
| Pravastatin | C10AA03 | 25.20 milligram |

**Supplemental Table 2 Septic shock risk and adjusted hazard ratios (aHRs) associated with statin use among patients with T2DM**

|  | **Crude HR (95%CI)** | | ***P* value** | **Adjusted HR (95%CI)^*^** | | ***P* value** |
| --- | --- | --- | --- | --- | --- | --- |
| **Stain users or nonusers** |  | |  |  | |  |
| Nonusers | **Reference** | | | | | |
| Statin users | 0.32 | (0.31, 0.33) | <0.0001 | 0.34 | (0.33, 0.35) | <0.0001 |
| **Different classes of statins** |  |  |  |  |  |  |
| Nonusers | **Reference** | | | | | |
| *Hydrophilic statins* |  |  |  |  |  |  |
| Pravastatin | 0.27 | (0.26, 0.29) | <0.0001 | 0.29 | (0.28, 0.31) | <0.0001 |
| Rosuvastatin | 0.28 | (0.27, 0.30) | <0.0001 | 0.31 | (0.30, 0.33) | <0.0001 |
| *Lipophilic statins* |  |  |  |  |  |  |
| Pitavastatin | 0.06 | (0.03, 0.08) | <0.0001 | 0.06 | (0.04, 0.10) | <0.0001 |
| Fluvastatin | 0.40 | (0.38, 0.41) | <0.0001 | 0.38 | (0.36, 0.40) | <0.0001 |
| Simvastatin | 0.30 | (0.29, 0.31) | <0.0001 | 0.34 | (0.33, 0.36) | <0.0001 |
| Lovastatin | 0.53 | (0.51, 0.55) | <0.0001 | 0.50 | (0.48, 0.53) | <0.0001 |
| Atorvastatin | 0.30 | (0.28, 0.31) | <0.0001 | 0.32 | (0.31, 0.33) | <0.0001 |
| **Cumulative dose of statins DDD per year** |  |  |  |  |  |  |
| Nonusers | **Reference** | | | | | |
| Q1 | 0.52 | (0.50, 0.54) | <0.0001 | 0.53 | (0.52, 0.55) | <0.0001 |
| Q2 | 0.36 | (0.35, 0.37) | <0.0001 | 0.39 | (0.38, 0.41) | <0.0001 |
| Q3 | 0.21 | (0.20, 0.23) | <0.0001 | 0.26 | (0.24, 0.28) | <0.0001 |
| Q4 | 0.12 | (0.11, 0.13) | <0.0001 | 0.14 | (0.13, 0.15) | <0.0001 |
| ***P* for trend** |  |  | <0.0001 |  |  | <0.0001 |

**Abbreviations**: aHR, adjusted hazard ration; HR, hazard ratio, CI, confidence interval; DDD, defined daily dose; T2DM, type 2 diabetes mellitus; Q, Quartile

* The aHR was derived from the inverse probability-weighted Cox model considering statin use as a time-dependent covariate, and the model was adjusted for age groups, sex, income levels, urbanization, types of antidiabetic drugs used, antidiabetic drugs, diabetic severity (aDCSI score), coexisting comorbidities, medication use, and CCI scores.

**Supplemental Figure 1. Kaplan****–Meier analysis of the cumulative curves of sepsis for statin users and nonusers in patients with T2DM**


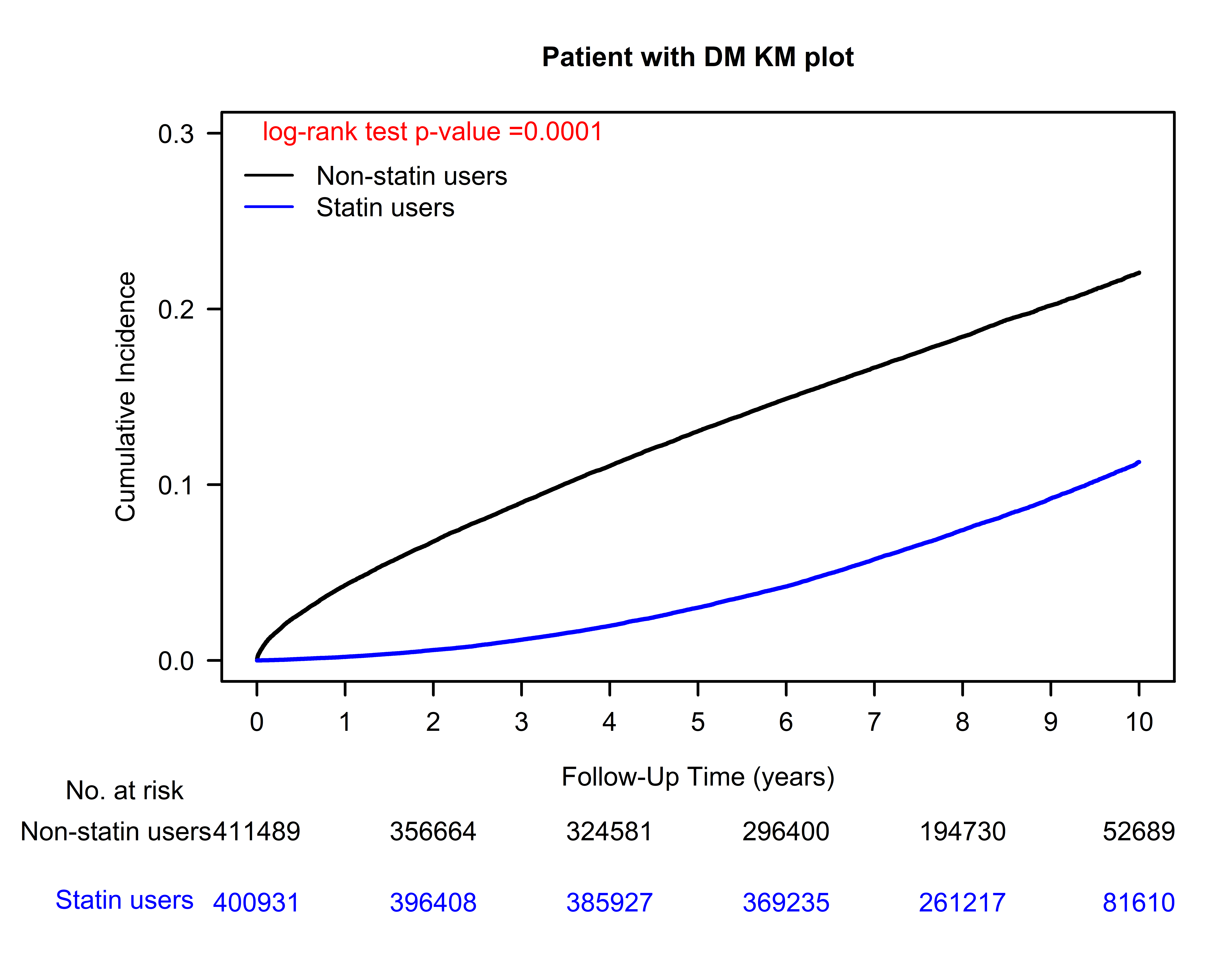


**Supplemental Figure 2 Intensity of statin use (DDD) and the hazard ratio of sepsis**


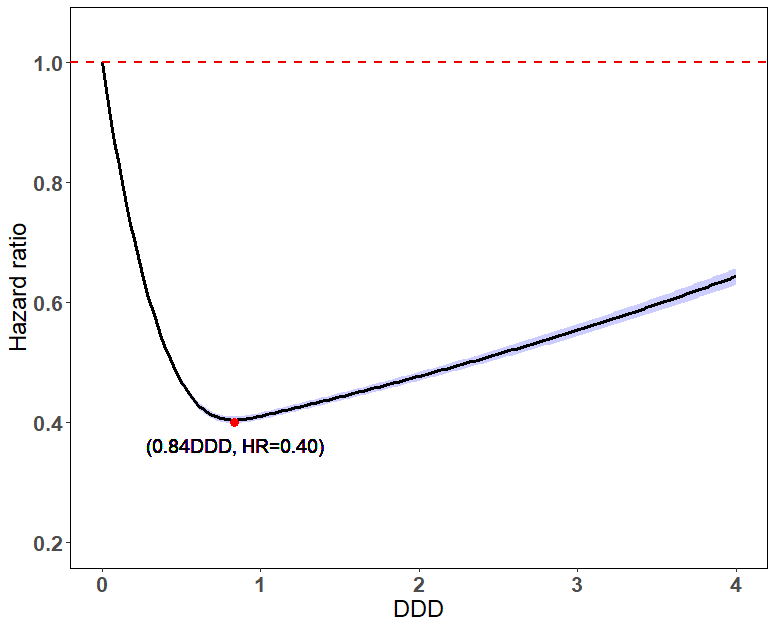


**Supplemental Figure 3 Study flow-chart**

≥40 years who had been diagnosed with type 2 diabetes between 2008 and 2020 (N=861,571)

Exclusion Criteria

1) Missing data on age at diagnosis or date of diagnosis (N=10,440)

2) Multiple classes of statins during the follow-up period (N=29,775)

3) Recurrent sepsis were excluded (N=8,936)

Type 2 Diabetes with or without statin use (N=812,420)

Inverse probability of treatment-weighted of age groups, sex, income levels, urbanization, types of antidiabetic drugs used, antidiabetic drugs, diabetic severity, coexisting comorbidities, Medication use related to the risk of sepsis, and the Charlson comorbidity index score

Stain Users (≥28 cDDD-year)

(N = 411,489)

Statin Nonusers (0 cDDD-year)

(N = 400,931)

Comparison Group

Case Group
